# Flour Making

**Published:** 1876-04

**Authors:** 


### FLOUR MAKING.

Now and then our grocer sends us a barrel of poor flour, from which good bread or light buscuit cannot be made. The flour appears to be well enough; cannot see that it differs in any perceptible particular from the former barrel from which the best of bread was obtained. But the Matron declares this particular barrel to be "good for nothing," and directs the grocer to exchange it for a better. *Why* flour should be good in one instance and poor in another, when it has all been obtained from one mill and manufactured from the same wheat, is what we endevored to ascertain. So, one day Mr. Holcomb, of the firm of Huntley, Holcomb & Heine, dealers in mill supplies, put in an appearance, and we set ourselves deliberately at work to "interview" him upon this subject, when we gleaned the following facts, which we thought good enough to print.

In the first place, we were informed that not one miller in ten understands how to grind flour. Well, that statement was sufficient to account for those nine poor barrels, without much further investigation. But we persevered, and learned still further, how difficult it is to keep the burrs in perfect order, so that good flour can be obtained. These burrs are made from stone possessing a peculiar grit and hardness, and are quarried in the southern part of Illinois, in Georgia, and in Missouri. The best in this country comes from Missouri; but the best of all, and which is used by our most successful flour makers, come from France. These stones are cut in a triangular shape, about fourteen inches wide at longest angle, and eight to ten inches thick, and are then manufactured into the burrs of about six feet in diameter, with which most of us are familiar.

The difficulty is to keep the faces of these stones perfectly even and level, in face with each other, and in *tram* with the spindle. If any high points exist upon the face of either stone, or the stones are not perfectly level with regard to each others' faces, they will touch, when the "life" is crushed out of the grain, and the flour becomes pasty and sticky. Great skill is requisite in the picking and adjusting of these stones, and as Mr. Holcomb remarked, "every dunce is not capable of doing it, although many mill owners think he is." If the stones run so close as to become heated, the flour is injured, and if not close enough, it is too coarse with which to make light bread.

After grinding, the next process of importance, is the bolting. This is accomplished through a very fine, silk cloth, manufactured in Switzerland for the purpose, is forty inches wide, and costs three dollars a yard. The cloth ordinarily used contains from ten to twenty-eight thousand meshes to the square inch, showing to what impalpable fineness our flour is reduced. The finer it is ground, however, without "pasting" it, i. e., by having the burrs run so close together as not to have their surfaces touch, the better it is.

Our best flour is obtained from the "middlings," which is the bran and unground particles of the wheat, which remain in the bolter after the first grinding is accomplished. This product was formerly sold as the poorest grade of flour, but now, after having passed through the "purifier," in which the bran and other deleterious qualities are removed, it is reground, rebolted, and then becomes our very best flour for bread-making.

Many other interesting points were elucidated by our well informed friend, but sufficient has already been written to show how necessary it is to have a good miller, upon whom depends almost entirely the good or bad qualities of our flour.

PREVENTION OF NAIL-BITING.—A correspondent of the *British Med. Jour.*, having asked for a cure for this habit, a reply was given, suggesting the application of styptic colloid, (tannin in collodion), in a thin line at the end of each nail. It dries quickly, forming a film, the bitter taste of which acts as a reminder. It must be re-applied, however, whenever the hands have been washed.

